# Leaf trait variation in species-rich tropical Andean forests

**DOI:** 10.1038/s41598-021-89190-8

**Published:** 2021-05-11

**Authors:** Jürgen Homeier, Tabea Seeler, Kerstin Pierick, Christoph Leuschner

**Affiliations:** 1 Plant Ecology and Ecosystems Research, University of Goettingen, Untere Karspüle 2, 37073 Goettingen, Germany; 2 Centre for Biodiversity and Sustainable Land Use, University of Goettingen, Büsgenweg 1, 37077 Goettingen, Germany

**Keywords:** Ecosystem ecology, Forest ecology, Tropical ecology, Plant ecology

## Abstract

Screening species-rich communities for the variation in functional traits along environmental gradients may help understanding the abiotic drivers of plant performance in a mechanistic way. We investigated tree leaf trait variation along an elevation gradient (1000–3000 m) in highly diverse neotropical montane forests to test the hypothesis that elevational trait change reflects a trend toward more conservative resource use strategies at higher elevations, with interspecific trait variation decreasing and trait integration increasing due to environmental filtering. Analysis of trait variance partitioning across the 52 tree species revealed for most traits a dominant influence of phylogeny, except for SLA, leaf thickness and foliar Ca, where elevation was most influential. The community-level means of SLA, foliar N and Ca, and foliar N/P ratio decreased with elevation, while leaf thickness and toughness increased. The contribution of intraspecific variation was substantial at the community level in most traits, yet smaller than the interspecific component. Both within-species and between-species trait variation did not change systematically with elevation. High phylogenetic diversity, together with small-scale edaphic heterogeneity, cause large interspecific leaf trait variation in these hyper-diverse Andean forests. Trait network analysis revealed increasing leaf trait integration with elevation, suggesting stronger environmental filtering at colder and nutrient-poorer sites.

## Introduction

Tropical montane forests (TMFs) provide valuable ecosystem services to the human populations in their neighborhoods, by supplying drinking water and wood, stabilizing slopes and contributing to climate warming mitigation through the sequestration of substantial amounts of carbon^1,2^. Tropical mountains often harbor a highly diverse and unique flora and fauna^3^, which makes them priority objects of conservation agendas. In many tropical regions, TMFs are threatened by conversion to pastures or agroforestry systems and logging, and their biota are particularly vulnerable to climate warming^4^. Elevated temperatures and a higher evaporative demand expose many mountain forest plants with adaptation to cool-humid climates to stress, which may result in reduced vitality and productivity^5^, and often upward movement of biota and the thermophilisation of TMF communities^6,7^. The consequences of these changes for ecosystem integrity and functioning are difficult to predict due to the high abiotic and biotic complexity of TMF landscapes, which change with topography and elevation over short spatial distances^3,8,9^.

In these highly diverse ecosystems, it is hardly possible to link the abundance or performance of single species to their contribution to ecosystem functioning. Screening species-rich communities for the variation in functional traits and their inter-relationships along environmental gradients may help to understand the abiotic drivers of plant performance and biotic interactions in quantitative terms and eventually could lead to predictive models of the response of species-rich forest ecosystems to environmental change^10–12^.

Global plant trait data sets show that important leaf functional traits are highly coordinated, which is reflected in a leaf economics spectrum^13,14^. Tree species can usually be arranged on an axis from acquisitive to conservative resource use strategies. Plants which follow an acquisitive strategy, should build leaves with relatively high N and P content and high photosynthetic capacity, while the foliage is less durable and more susceptible to herbivore attack. In contrast, plants with a conservative strategy should maintain leaves with relatively low N and P content and a lower photosynthetic activity, but higher resistance against herbivores and greater longevity^13^.

With increasing elevation in mountains, decreasing temperatures and soil fertility expose plants to an increasingly unfavorable environment as reflected by less species-rich and less productive forests^15–17^. This should filter out species lacking appropriate adaptation. It is plausible that interspecific trait variation in the community declines with increasing elevation due to the reduction in plant diversity as a result of environmental filtering by increasingly harsh growing conditions. Empirical evidence supporting this assumption in tropical forests is scarce.

Trait variation in communities is greatly enhanced by intraspecific variability, the importance of which for tree survival in changing environments has only recently been fully recognized^18^. High phenotypic variability due to genetic variation within populations and the resulting trait plasticity can be decisive for a population to survive in stressful environments. Although trait-based studies have improved our understanding of environmental drivers, there is a lack of studies linking interspecific and intraspecific components of trait variation in forest communities along gradients of resource availability^19–21^. Intraspecific variation can contribute a considerable proportion of total trait variation in plant communities^22–26^.

Another important factor determining plant leaf trait syndromes is tree evolutionary history. Several regional to global-scale studies found that evolutionary constraints, i.e. phylogeny, strongly impact leaf trait variation, an effect that in most traits is stronger than the influence of environmental factors^24,27–29^.

Most plant traits are multifunctional and connected to other traits through complex direct and indirect interactions, and the study of plant trait networks is a promising new tool to understand plant adaptation to their environment^30–32^. Trait constellations and network topologies differ among plant communities, and they thus may shift along gradients of resource availability and with changes in species composition^32^. The analysis of plant trait networks along environmental gradients at the local scale may also be a straightforward approach to understand the importance of different traits for plant adaptation within a given community^33^. The integration of plant traits in trait networks, i.e. the covariance between multiple traits among species, is driven by the biophysical, evolutionary and environmental constraints acting in a given habitat^34^. Towards more stressful environments, trait integration should increase, because species are coordinating multiple traits for optimizing resource conservation, whereas under more favourable conditions, broader variation in independent trait combinations is possible, which may result in weaker trait integration^34,35^.

We measured twelve leaf traits of 52 tree species in old-growth Andean forests along an elevation transect from 1000 to 3000 m a.s.l. in southern Ecuador and determined both interspecific (community-level) and intraspecific (species-level) trait variation. Traits studied were leaf size, specific leaf area (SLA), leaf thickness, leaf toughness, leaf dry matter content (DMC), and foliar nutrient contents (N, P, Ca, K, Mg, Al) and element ratios (N/P). These traits are related to photosynthetic carbon gain (foliar N and P, leaf thickness), the costs of light interception and CO_2_ assimilation (SLA and foliar nutrient relations), and herbivore susceptibility (leaf toughness, foliar N). The species selected were either common taxa in the respective communities or they represented characteristic tree life strategies of the three elevation levels.

We tested the following hypotheses: (1) Leaf size, SLA and foliar N and P decrease with elevation, while leaf thickness and toughness increase, indicating a trend toward more conservative resource use strategies at higher elevations, (2) interspecific trait variation decreases with elevation, (3) the phylogenetic effect on trait variation is stronger than the effect of elevation, (4) the contribution of interspecific trait variation to the community’s response to the environment is generally larger than that of intraspecific variation, and (5) trait integration is increased towards higher elevation where environmental conditions are harsher.

## Results

### Dependence of leaf traits on elevation and phylogeny

**Figure 1:**
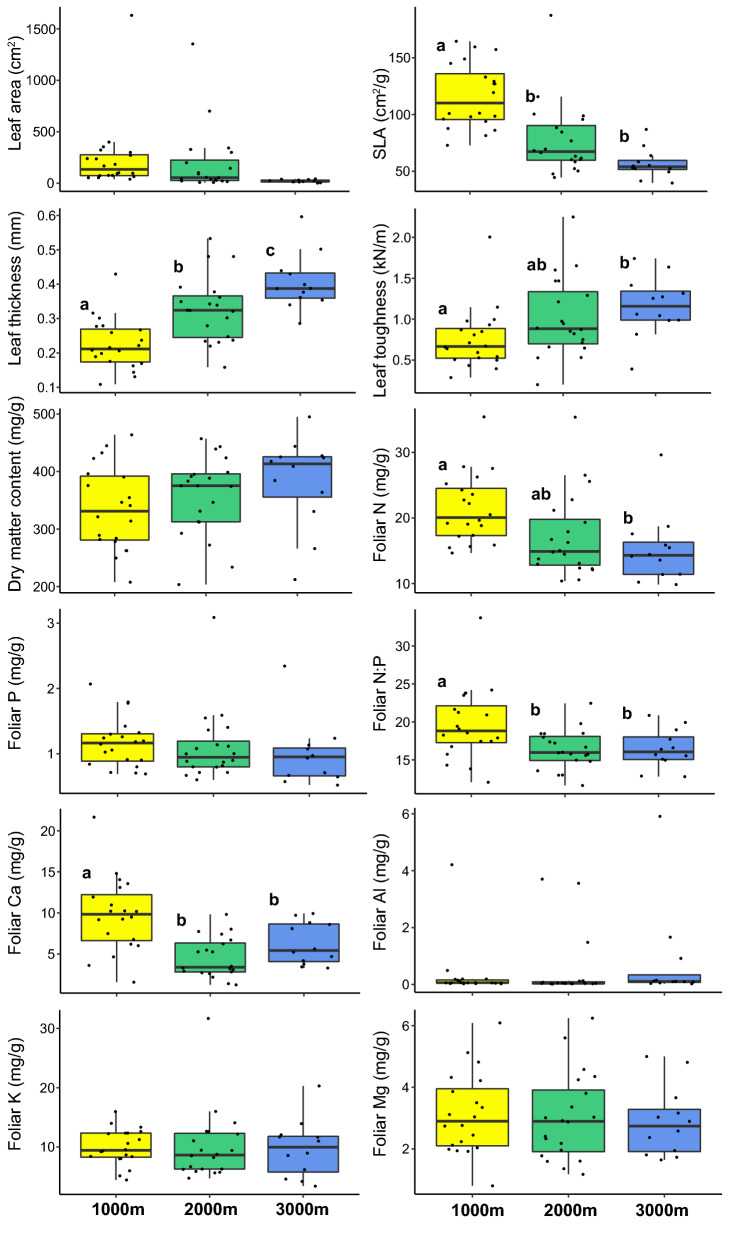
Boxplots showing the leaf trait distribution of the studied tree species at the three elevations. Different letters represent significant differences between study sites (Tukeys HSD-test).

Six of the 12 studied leaf traits varied significantly between the three elevational levels (Fig. 1). The elevation-level means of SLA, foliar N and Ca, and foliar N/P ratio of the studied species decreased from 1000 to 3000 m, while dry matter content and leaf thickness and toughness increased.

**Figure 2:**
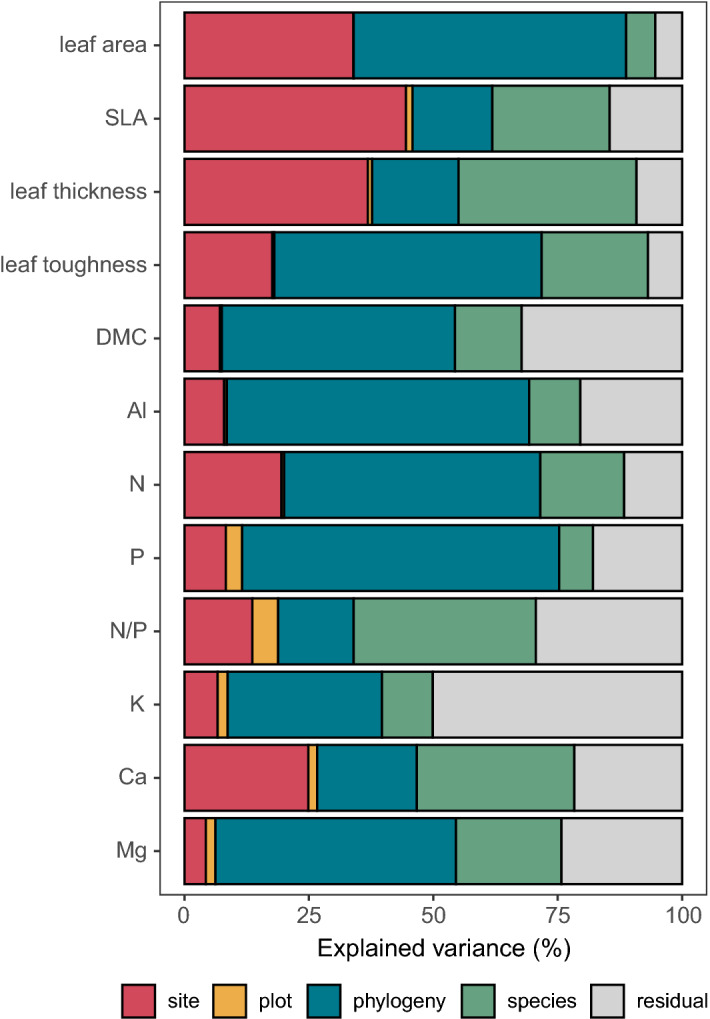
Fractions of variance explained by the components of the Bayesian mixed models for the 12 studied leaf traits.

The total variance explained by fixed and random factors in the Bayesian mixed models ranged between 49.9% (K) and 94.6% (leaf area) (Fig. 2, Appendices A4 and A5). The analysis of trait variance partitioning with Bayesian mixed models revealed for most traits (leaf area, leaf toughness, DMC, and foliar N, P, Mg and Al contents) a dominant influence of phylogeny, except for SLA, leaf thickness and foliar Ca, where elevation had the largest influence. The phylogenetic influence is particularly visible in foliar Al, where the highest levels were found in Melastomataceae (*Graffenrieda*, *Miconia*, *Meriania*). The explanatory power of the phylogenetically independent species effect was particularly large in case of leaf thickness and foliar N/P ratio. The variance explained by plot, i.e. small-scale edaphic variation, was generally very small. More than 30% of the variance was unexplained in case of foliar K content, N/P ratio and dry matter content.

### Intra- and interspecific trait variation

**Figure 3:**
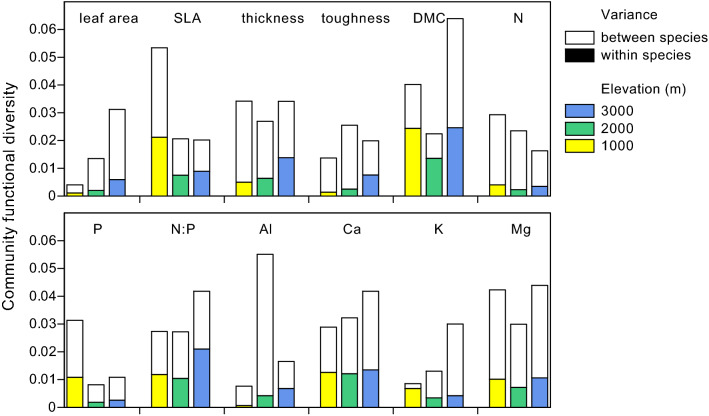
Partitioning of community functional diversity into between- and within-species functional diversity for the 12 studied leaf traits at the three elevation levels.

Community functional diversity and the contribution of within-species and between-species variance differed largely between the studied 12 major foliar traits and between elevation levels (Fig. 3, Appendices A5 and A6). For leaf area, DMC, and foliar Ca and K concentrations, community functional diversity increased from 1000 to 3000 m, while it was highest at 1000 m for SLA, and foliar N and P concentrations.

For most traits, intraspecific variation tended to be lower than interspecific variation in our sample. In case of SLA, DMC, foliar N/P ratio and foliar Ca, however, within-species variation contributed substantially to community functional diversity (> 30% at the three elevation levels). The intraspecific variance component was particularly high in case of DMC at 1000 m and 2000 m (61%) and foliar K at 1000 m (80%).

There was no clear overall trend in intra- and interspecific trait variation with elevation.

### The leaf trait space and trait networks

**Figure 4:**
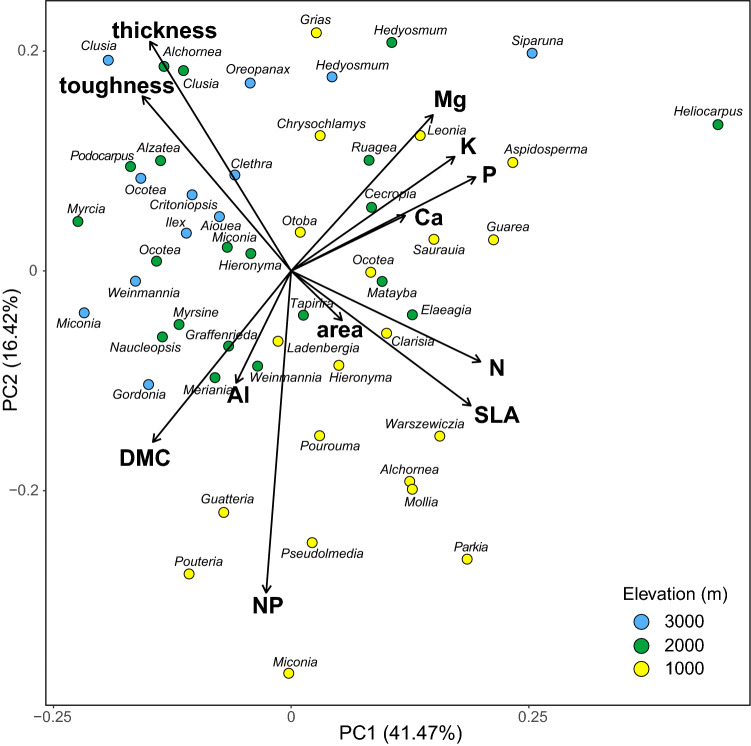
Principle components analysis (PCA) showing the distribution of the 52 tree species from the three study sites in the leaf trait space.

The principal components analysis shows a relatively clear separation of the species at 1000 m from those at 2000 m and 3000 m based on their leaf traits (Fig. 4). The separation between the species at 2000 m and 3000 m was less distinct. The first principal component, which explains 41% of the variance, represents leaf N, P and SLA (all with positive loadings; see Appendix A7), and toughness, thickness and DMC (all with negative loadings). It represents a resource acquisition axis separating conservative (tough, thick leaves with high DMC) and upper elevation species from acquisitive (with thin leaves and high nutrient content) and lower elevation tree species. The second axis, explaining 16%, is mainly related to leaf thickness, toughness and Mg content (all with positive loadings), and N/P ratio, DMC and SLA (all with negative loadings).

**Figure 5:**
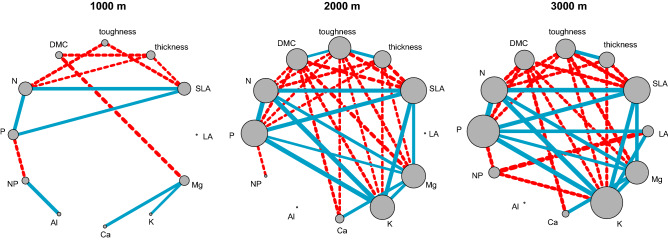
Leaf trait networks for the three study sites (from left to right: premontane, lower montane and upper montane forest). Only significant correlations (*p* < 0.05) are shown. Solid blue lines and dashed red lines show positive and negative correlations, respectively. Line thickness represents the strength of the correlations. The size of the trait nodes gives the sum of the weights of edges linked to a focal node (“node strength”).

The analysis of trait networks (Fig. 5) indicates that the interconnectedness of the 12 leaf morphological and chemical traits increases with elevation. This is supported by increasing trends of edge density and average clustering coefficient and a decreasing trend of average path length in the trait networks from lower (1000 m) to higher (2000 and 3000 m) elevations (Table 1).

**Table 1:** Parameters characterizing the studied leaf trait networks at the three study sites (see Fig. 5).

	1000 m	2000 m	3000 m
Edge density	0.197	0.470	0.470
Average path length	3.0	1.333	1.491
Average clustering coefficient	0.375	0.855	0.724

The apparent functional importance of foliar nutrients (N, P, K, Mg) increased toward higher elevations, as indicated by the assessment of network centrality (Appendix A8). Leaf area (at 1000 m and 2000 m) and foliar Al (at 2000 m and 3000 m) are not integrated in the networks, since both traits had no significant correlation to any other trait in the communities of the respective elevations.

## Discussion

### Elevational change in leaf trait spectra and possible drivers

Along the mountain slope from 1000 to 3000 m, we found a significant decrease in mean SLA and foliar N content, while leaf thickness and toughness increased. Leaf Ca and N/P ratio both decreased significantly from 1000 to 2000 m and remained constant higher upslope. The elevational trends in SLA and foliar N are in line with the results from other tropical elevation gradients^36–38^. Thus, the average tree leaf in the sun canopy of the upper montane forest is thicker and tougher, and contains less N than leaves in the premontane forest. This points at progressive growth limitation primarily through N shortage with increasing elevation, while the influence of P limitation seems to be unrelated to elevation. Leaf N/P ratios above 16 for most tree species regardless of elevation indicate the limiting role of P for tree growth in the study area^39^. N and P fertilization experiments at the study sites^40,41^, the observed large increase in tree root/shoot ratio along the slope^42^, and in situ measurements of nitrogen mineralization rate and P availability in the soil^43^ support this assumption. One plausible explanation is that the temperature decrease is hampering soil biological activity, thereby reducing organic matter decomposition and mineralisation rate. As N supply is more dependent on soil biological activity than P supply, which is largely affected by parent rock and fixation processes in the soil, increasing N limitation and a decreasing foliar N/P ratio at higher elevations were expected. The leaf trait changes along the slope imply that temperature is acting mainly through N and P availability as an environmental filter, sorting species according to their leaf traits from more acquisitive to more conservative at higher elevations. This supports our first hypothesis. Reduced SLA and higher leaf toughness must be interpreted primarily as an adaptation to increase nutrient use efficiency through an extension of leaf life span, and not as an investment in more defence, as leaf herbivory in Andean forests decreases with elevation^44^. Further, acquisitive traits are less an advantage in environments, where competition for light is less intense, as in the upper montane forest.

### Trait variation and environmental filtering

At all three forest sites, certain species maintained much higher foliar N and P contents (> 28 mg N g^−1^ and > 2.2 mg P g^−1^) than the community mean, notably *Parkia *sp. at 1000 m as a N_2_-fixing legume, *Heliocarpus americanus* at 2000 m, and *Siparuna muricata* at 3000 m. *Heliocarpus* and *Siparuna* differed from the bulk of species also in terms of their hygromorphic foliage (low DMC and high SLA) and low leaf toughness, i.e. clearly acquisitive leaf traits. On the other hand, species with conservative traits are also present at lower elevation, e.g. the small-stature trees *Grias peruviana* (Lecythidaceae) and *Chrysochlamys* *membranacea* (Clusiaceae) at 1000 m, and *Myrcia* sp. nov. (Myrtaceae), *Myrsine coriacea* (Myrsinaceae) and *Podocarpus oleifolius* (Podocarpaceae) at 2000 m. This points at a considerable diversity in resource acquisition and utilization strategies among the species in the premontane, lower montane and upper montane forest. The occurrence of a few N_2_-fixing Fabaceae cannot explain this functional variance, as the *Parkia* species occurs only at 1000 m.

In the majority of traits, community-level variation was lowest at 3000 m, but for some foliar nutrients (N, P, K), it was lowest at 1000 m. Hence, our hypothesis (2) was not fully confirmed.

We thus conclude that filtering through an increasingly cold and nutrient-limited environment seems to be only partly shaping the functional trait composition of these communities. In addition, small-scale heterogeneity in soil physical and chemical conditions, enhanced through topographic gradients in the rugged mountain terrain, is one possible factor that might allow maintaining a higher trait variability also at higher elevations, as it creates different edaphic niches at short distance^8,43,45^. Yet, environmental filtering apparently has increased the integration of leaf traits in higher-elevation trees, as is suggested by the trait network analysis. This indicates that the tree community responds with a more efficient acquisition and mobilization of resources^30,32,34,35^. Even if the colder and less fertile site conditions did not reduce interspecific trait variation in all leaf traits, they seem to have restricted the degrees of freedom of possible trait combinations. When ascending from 1000 to 3000 m, the foliar nutrients (N, P, K, Mg) gain in importance (network centrality) and trait connections become stronger (hypothesis 5), which underpins the key role nutrient limitation is playing for plant growth and survival in these environments. Although the 2000 m site has the same elevational distance to the two other sites, this tree community is more similar in its leaf functional properties and leaf trait coordination to the community at 3000 m than at 1000 m. A plausible explanation is the shared parent rock material at the two uppermost sites (metamorphic schists and sandstones), while granodioritic rocks are found at 1000 m^43^.

### Inter- and intraspecific trait variation and the role of phylogeny

Our variance partitioning analysis demonstrates a great importance of phylogeny on the variation of many leaf traits in these species-rich forests, largely supporting our third hypothesis. With the inclusion of a needle-leaved conifer (*Podocarpus oleifolius*), leaf-succulent *Clusia* species, Al-accumulating Melastomataceae species and various Lauraceae species, i.e. early-diverged laurophyllous angiosperms from the Magnoliid clade, our sample of 52 species from 43 genera and 32 families covers very different leaf habits, which are controlled by conservatively inherited traits.

We found leaf size, leaf dry matter content, and leaf toughness, i.e. cell wall- and cuticle-related properties, and foliar P, N, Mg, Al and K contents to be largely dependent on phylogeny. These traits apparently vary less with climate and soil conditions than SLA and the associated lamina thickness, which can rapidly be modified via light, water and nutrient effects on mesophyll cell growth^46^.

The low phylogenetic effect on foliar N/P ratio suggests that the different species operate in a rather narrow leaf stoichiometric window. A considerable part of the unexplained (residual) variance in the foliar element content traits may relate to variation in local soil conditions and the evaporative demand, which will influence the foliar accumulation especially of mobile nutrients such as K^47^.

We found for most of the 12 studied canopy leaf traits a smaller intraspecific variation than interspecific variation in the studied communities, which supports our hypothesis (4) and is in line with the results of^36^. Sampling more replicates on the species level and including additional less common tree species might change this relation, as rare tropical tree species have been shown to exhibit higher intraspecific trait variation than common species^48^. Based on our 52-species sample, we conclude that leaf trait variation is greater between species than within species in these highly diverse forest communities. This is a consequence of the rarity of most species, allowing for only a relatively small trait variation at the population level, where selection processes can act on. Since intraspecific trait variation is the basis for adaptation at the population and species level, the highly diverse Andean forests are more susceptible to environmental change and related shifts in species composition than less species-rich forests elsewhere. Intraspecific trait variation showed no systematic decrease with elevation in most traits. Tree species in harsher environments thus seem to maintain a similar trait plasticity as populations growing under more favourable conditions.

## Conclusions

Our in-depth study of 12 leaf traits in 52 tree species of species-rich tropical Andean forests demonstrates the high interspecific variation in leaf functionality that is introduced in these communities primarily through phylogenetic diversity. In the majority of traits, more than two third of total variance is contributed by interspecific variation, only a third or less by intraspecific variation. Even though our species sample covers only about five percent of the regional tree flora and thus may have missed certain tree genera and species with specific traits, we selected the bulk of the more abundant species and thus those taxa, which likely have a large influence on ecosystem functioning. Future studies in the remaining, mostly rare species of the communities have to reveal the contribution of these taxa to community functional diversity in the studied tropical montane forests. The minor relevance of intraspecific trait variation at the community level contrasts with the situation in species-poor extra-tropical forests, where tree species show large leaf trait variation within populations, which forms the basis for successful population-level adaptation. Despite significant deterioration of thermal and soil chemical growing conditions between 1000 and 3000 m elevation, we did not find an elevational trend towards reduced interspecific trait variation in the upper montane forest. Yet, the shift in tree species composition from the dominance of a more acquisitive strategy with higher photosynthetic capacity to a more conservative life strategy with greater leaf longevity and the more efficient acquisition and mobilization of resources, as indicated by increasing connectedness in the leaf trait networks, demonstrate, how environmental filtering is shaping the tree species composition of highly diverse tropical Andean forests.

## Materials and methods

### Study sites and examined tree species

The study was conducted at three sites in the Andes of southern Ecuador along an elevation gradient at ca. 1000 m (Bombuscaro, Podocarpus NP), ca. 2000 m (San Francisco Reserve) and ca. 3000 m elevation (Cajanuma, Podocarpus NP) in the Provinces of Loja and Zamora-Chinchipe. All sites are located in protected forest areas. At each elevation three permanent 1-ha plots were established in 2018, choosing representative portions of old-growth forest without visible signs of human disturbance (Appendix A1).

The forest types at the three sites differ in floristic composition, species richness and structural characteristics^49^: The premontane rain forest (below 1300 m) at the lowermost site reaches 40 m in height with common tree families being Fabaceae, Moraceae, Myristicaceae, Rubiaceae, and Sapotaceae. It is replaced at 1300–2100 m by smaller-statured lower montane rain forest with Euphorbiaceae, Lauraceae, Melastomataceae, and Rubiaceae as characteristic tree families, and above 2100 m by upper montane rain forest with a canopy height that rarely exceeds 8–10 m. Dominant tree families of the latter forest type are Aquifoliaceae, Clusiaceae, Cunoniaceae, and Melastomataceae. Tree species turnover is complete between premontane and upper montane forest, while a few tree species are shared between lower montane and premontane or upper montane forest types.

The climate is tropical humid with a precipitation peak from June to August and a less humid period from September to December. Mean annual temperature decreases with elevation from 20 °C at 1000 m to 9.5 °C at 3000 m, while annual precipitation increases from around 2000 mm at the two lowermost sites to 4500 mm at 3000 m. Typically, there are no arid months with < 100 mm precipitation^50,51^.

The study sites are characterized by relatively nutrient-poor soils on metamorphic schists and sandstones (2000 and 3000 m) or granodioritic rocks (1000 m)^43^. Soils are slightly more fertile on lower slope positions and at lower elevations than on upper slopes and at upper elevations^43,52^. The decreasing nutrient availability is reflected by decreasing forest biomass and productivity with increasing elevation^16,43,53^, and along the topographical gradient from lower to upper slope position^8^.

We selected 52 tree species in total, 20 tree species each from the species-rich premontane and lower montane forests, and twelve species from the upper montane forest. The species included the most abundant tree species in the permanent 1-ha plots at each elevation and in addition represented the major occurring tree life strategies from each study site, covering fast-growing pioneers to late-successional tree species and understory species to tall canopy trees (Appendix A2). In addition, we used available data on SLA and wood specific gravity (WSG) from previous studies^54^ to select species at the three sites that covered the known range of these two functional traits. The selected species represent 41.0% (at 1000 m), 50.3% (at 2000 m) and 32.0% (at 3000 m) of the total tree basal area (trees ≥ 10 cm dbh) of the three study plots at the respective elevation.

### Leaf sampling and trait analyses

We sampled 8–10 replicate trees per species, randomly selected from the known individuals within the three 1-ha plots (for four species only 5–7 appropriate tree individuals were found). In total 421 trees were sampled.

The sampling campaign took place from February to March 2019. We collected 2–3 branches per tree from the top of the crown with as much sun exposure as possible with all attached leaves and took them in sealed polyethylene bags (filled with water-soaked tissues) to the research station, where further processing took place. The branches were placed over night in water to achieve water saturation of the leaf tissue prior to the measurements.

Per tree, 20 young but fully developed sun leaves without signs of herbivory were stripped from the branches and used for the analyses. We took care that all leaves were fully expanded and not senesced and attempted to cover the full range of leaf sizes present. Three additional leaves of average size were taken to quantify leaf thickness and toughness. In case of compound leaves, we measured the morphology of the individual leaflets. In a few species with particularly large leaves (e.g. *Cecropia, Graffenrieda,* and *Pourouma*), a smaller number of leaves was investigated due to time constraints during optical leaf area determination.

For determining average leaf size (one-sided leaf area, LA, cm^2^), the fresh leaves including petioles were scanned in color (Canon LIDE 100, 150 dpi). LA was determined from the scanned leaf silhouettes with the software WinFOLIA 2014a (Régent Instruments, Quebec, QC, Canada). Subsequently, leaf fresh weight was determined and the leaves dried at 60 °C for at least three days to determine leaf dry weight and foliar water content. Leaf dry matter content (DMC, mg g^−1^) was calculated as the quotient of leaf dry weight to leaf fresh weight. Specific leaf area (SLA, cm^2^ g^−1^) was calculated by dividing total LA by total leaf dry mass.

Leaf thickness (mm) was measured on three fresh leaves per tree with a digital micrometer (Mitutoyo M293-240-70, Mitutoyo Germany Ltd, Neuss, Germany) at each two locations on both sides of the main leaf vein in the middle between the secondary veins; measurements were subsequently averaged.

Leaf toughness (kN m^−1^) was estimated as the mean of six punch tests using a digital penetrometer (flat-ended 2.0 mm diameter steel punch, DS-50 N, Imada Inc., Japan) on three fresh leaves (excluding the midrib and other major veins) from each tree^55^.

Leaf dry mass was analyzed for its C and N content with a CN elemental analyzer (Vario EL III, Hanau, Germany), and for its P, Ca, K, Mg and Al content by ICP analysis (Thermo Scientific iCAP 7000 ICP-OES, Thermo Fisher Scientific, Germany) after HNO_3_ digestion of the ground leaf material.

### Statistical analyses

We compared trait means across the three elevation levels with Tukey’s HSD test.

The phylogenetic relationships of the studied tree species were extracted from the mega-tree of vascular plants ‘GBOTB.extended.tre’ using the R package V.PhyloMaker^56^ (Appendix A3). We used the R package brms^57^ to fit 12 Bayesian phylogenetic multilevel models^58^ with identical structure to describe the effect of the elevational level on the 12 investigated leaf functional traits. All leaf traits were log-transformed in order to handle skewness and heteroscedasticity. The observational units were the tree individuals. The models had the following structure:

$$\begin{aligned} & {\text{log}}\left( {Y_{i} } \right) \sim {\text{Normal}}\left( {\mu_{i} ,\sigma } \right) \\ & \mu_{ijk} = \alpha_{0} + \beta \cdot site_{i} + \alpha_{plot\left[ j \right]} + \alpha_{species\left[ k \right]} \\ \end{aligned}$$

where the response variable $Y$, a log-transformed leaf trait, is described by a normal distribution with the varying mean $\mu$ and a standard deviation of $\sigma$. The predicted value $\mu$ for observation $i$ in plot $j$ and of species $k$ is described by the intercept $\alpha_{0}$, a fixed effect for the categorical predictor site with three levels, a random intercept for plot $j$ ($\alpha_{plot\left[ j \right]}$) and a random intercept for species $k$. Whereas the plot effects were standard random effects as in usual mixed models, the random intercepts for the species were a combination of two components: a phylogenetic effect that incorporated the phylogenetic non-independence of residuals (called ‘phylogeny effect’ in short) and an phylogenetically-independent species effect that accounted for additional variance among species (called ‘species effect’ in short). The random effects were fit as following:

Random plot effects

$$\alpha_{plot\left[ j \right]} \sim {\text{Normal}}\left( {0,\tau_{plot} } \right)$$

Random species and phylogeny effects

$$\alpha_{species\left[ k \right]} \sim {\text{MVN}}\left( {0,\Sigma_{phyl} } \right)$$

$$\Sigma_{{phyl\left[ {m,n} \right]}} = \left\{ {\begin{array}{*{20}l} {\tau_{phyl}^{2} + \tau_{ind}^{2} } \hfill & {\quad {\text{if}}\;m = n} \hfill \\ {\tau_{phyl}^{2} \,\rho_{{phyl\left[ {m,n} \right]}} } \hfill & {\quad {\text{else}}} \hfill \\ \end{array} } \right.$$

For the random plot effect, a standard deviation parameter $\tau_{plot}$ was estimated as in usual mixed models. The two-component random effect on the species level was estimated using two variance parameters, $\tau_{phyl}^{2}$ for the ‘phylogeny effect’ and $\tau_{ind}^{2}$ for the ‘species effect’. The overall effect described by a multivariate normal distribution with a covariance of $\Sigma_{phyl}$. $\Sigma_{{phyl\left[ {m,n} \right]}}$ is a matrix with $\tau_{phyl}^{2} + \tau_{ind}^{2}$ on the diagonal and $\tau_{phyl}^{2}$ times the correlation $\rho_{{phyl\left[ {m,n} \right]}}$ derived from the phylogenetic variance–covariance matrix between the two species $m$ and $n$ elsewhere.

This model structure can be interpreted analogously to a classical PGLS model but offers the advantages of handling several observations per species instead of working with species means, and the possibility to include further random effects and comprehensive Bayesian inference^58^.

Weakly informative normal priors were used for the slope and intercept parameters. All variance components were assigned to weakly informative half-t priors. Models were fit with Hamiltonian Monte Carlo (HMC^59^) via the Stan probabilistic programming language^60^ using R package brms^49^. Sampling was performed for 10,000 iterations after a warmup of 10,000 iterations. The settings used for the HMC algorithm were an adapt_delta value (target acceptance rate) of 0.99 and a maximum tree depth of 15. The posterior distributions of the parameter estimates were summarized by the posterior mean and the 95% highest posterior density intervals (HDI^61^). Effects were considered credibly different from zero, when the 95% HDI did not include zero. The contribution of different model components to the total variance in the data was decomposed based on the approach of^62^, extended to a multi-level context analogous to^63^.

We followed the method proposed by de Bello et al.^64^ for partitioning of quadratic entropy with the Rao index to decompose total community variance into between-species and within-species effects. The R function ‘RaoRel.r’^64^ was used to compute the variance weighting by relative species abundance. Species relative abundance was based on the species’ average contribution to plot basal area in the three permanent 1-ha plots at the respective elevation level. For the analyses, we standardized each trait by dividing the values by the range of possible values for this trait^64^.

A principal components analysis (PCA) was used to summarize the correlation structure of the 12 standardized mean trait values for the 52 tree species at the three study sites.

We applied network analyses to assess the connectivity between the studied traits at each elevation level. For each study site, we calculated a matrix of trait-trait relationships using Pearson correlations. To avoid considering spurious correlations among traits, only statistically significant correlations (*p* < 0.05) were included^32,33^. Then, an adjacency matrix was created by assigning below threshold as 0 and above threshold as the integer of |r|× 10; thus, the adjacency matrix only shows the weighted presence or the absence of connections between pairs of plant traits. Finally, trait networks were visualized and informative parameters calculated with the R package igraph^65^.

In the constructed networks, traits are represented as nodes and their correlations are represented as the edges linking them. We used “degree”, “node strength”, and “betweenness” as indicators of network centrality. The degree is the number of edges that connects a focal node to other nodes; node strength is the sum of the weights of edges linking a focal node to adjacent nodes, and betweenness gives the number of shortest paths from all nodes to all others passing through the focal node.

To characterize the overall networks, we used “edge density”, “average path length” and “average clustering coefficient”. Edge density defines the proportion of present edges among nodes out of all possible edges in the network; average path length is the average "degrees of separation" between all pairs of nodes in the network, and the average clustering coefficient measures the probability that the adjacent nodes of a node are connected.

All statistical analyses were performed using the R v4.0.2 programming environment (https://www.r-project.org/)^66^.

## Supplementary Information

Supplementary Information

## Data Availability

All data supporting the results are archived under http://www.tropicalmountainforest.org/ (DOI 10.5678/lcrs/for2730.dat.1926).
