# Supplementary material for: Leaf trait variation in species-rich tropical Andean forests
Source: Sci Rep. 2021 May 11;11:9993. doi: 10.1038/s41598-021-89190-8 (PMC8113502; doi:10.1038/s41598-021-89190-8)
Supplement: Supplementary file 1 — Supplementary Information [file 41598_2021_89190_MOESM1_ESM.pdf]

Leaf trait variation in species-rich tropical Andean forests

Jürgen Homeier, Tabea Seeler, Kerstin Pierick & Christoph Leuschner

Supplementary Material

Appendix A1: Map of the study sites

Appendix A2: The 52 tropical montane forest tree species studied and their ecological characteristics.

Appendix A3: Phylogenetic tree of the study species.

Appendix A4: Model results

Appendix A5: Intraspecific trait variation at the three study sites, tree species are ordered by their mean values and genus names of species with high or low values are given.

Appendix A6: Results of functional diversity partitioning into between- and within-species effects.

Appendix A7: PCA results.

Appendix A8: Leaf trait network characteristics at the three study sites.

Supplementary Material

Appendix A1: Map of the study sites (modified after [40]).

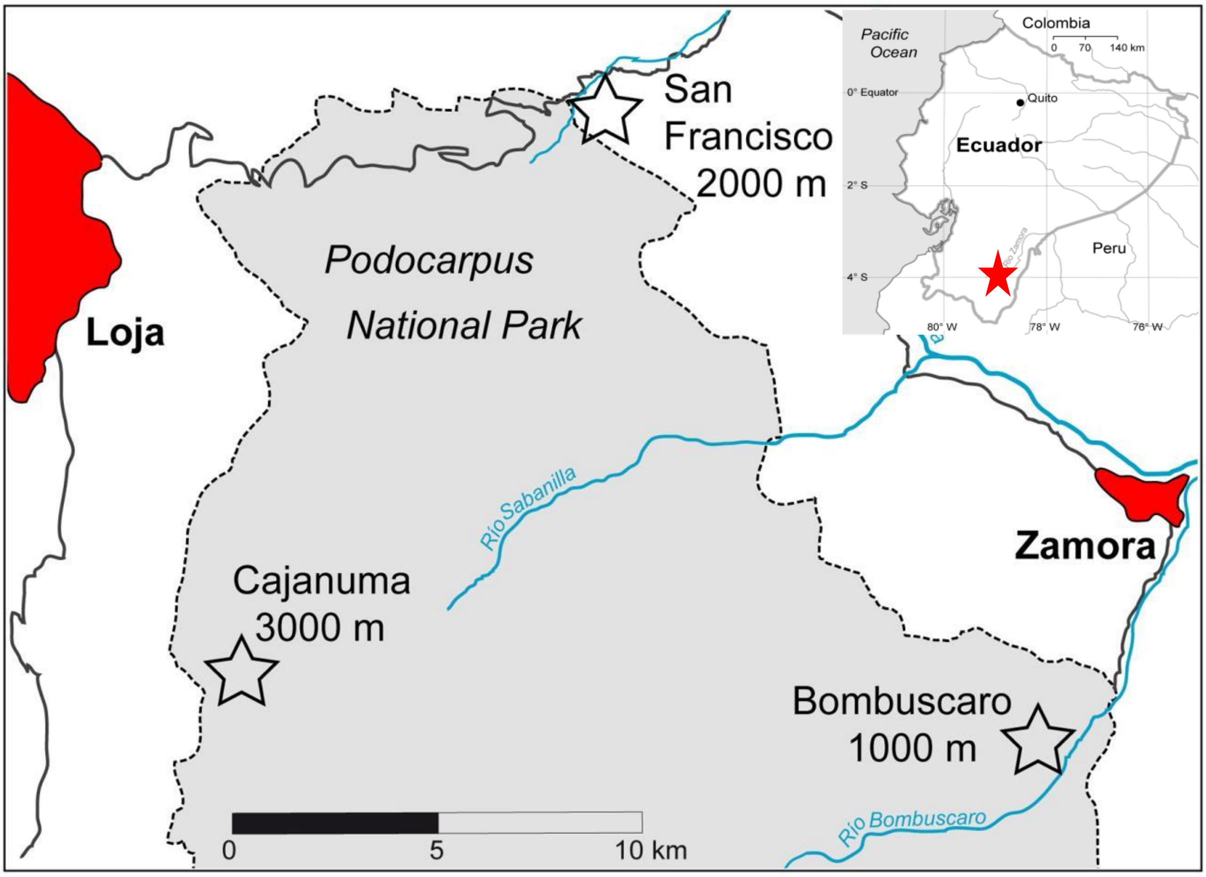

Appendix A2: The 52 tropical montane forest tree species studied and their ecological characteristics.

**Study site**	**Family**	**Species**	**trees**	**Successional stage** (**E**arly, **I**ntermediate, **L**ate)	**Life strategy** (**A**cquisitive, **I**ntermediate, **C**onservative)	**Size** (**U**nderstory, **I**ntermediate, **C**anopy)
1000	Actinidiaceae	*Saurauia sp1*	8	E	A	U
1000	Annonaceae	*Guatteria pastazae*	6	I	I	C
1000	Apocynaceae	*Aspidosperma rigidum*	8	L	C	C
1000	Clusiaceae	*Chrysochlamys*	8	L	C	U
1000	Euphorbiaceae	*Alchornea glandulosa*	9	E	A	I
1000	Fabaceae	*Parkia sp1*	8	I	I	C
1000	Lauraceae	*Ocotea sp1*	8	L	C	C
1000	Lecythidaceae	*Grias peruviana*	8	L	C	U
1000	Malvaceae	*Mollia gracilis*	7	E	A	C
1000	Melastomataceae	*Miconia aff punctata*	8	E	I	U
1000	Meliaceae	*Guarea macrophylla*	8	I	I	U
1000	Moraceae	*Clarisia racemosa*	8	L	C	C
1000	Moraceae	*Pseudolmedia laevis*	8	I	I	C
1000	Myristicaceae	*Otoba parvifolia*	8	E	A	C
1000	Phyllanthaceae	*Hieronyma oblonga*	8	I	I	C
1000	Rubiaceae	*Ladenbergia oblongifolia*	8	I	I	C
1000	Rubiaceae	*Warszewiczia coccinea*	8	L	I	I
1000	Sapotaceae	*Pouteria torta*	8	L	C	C
1000	Urticaceae	*Pourouma cecropiifolia*	6	E	A	I
1000	Violaceae	*Leonia crassa*	8	L	C	C
2000	Alzateaceae	*Alzatea verticillata*	9	I	I	C
2000	Anacardiaceae	*Tapirira guianensis*	9	E	A	C
2000	Chloranthaceae	*Hedyosmum goudotianum*	8	E	A	U
2000	Clusiaceae	*Clusia ducuoides*	8	L	I	C
2000	Cunoniaceae	*Weinmannia microphylla*	8	L	I	I
2000	Euphorbiaceae	*Alchornea lojaensis*	9	L	I	C
2000	Lauraceae	*Ocotea andina*	9	L	I	C
2000	Malvaceae	*Heliocarpus americanus*	8	E	A	I
2000	Melastomataceae	*Graffenrieda emarginata*	8	I	I	I
2000	Melastomataceae	*Meriania franciscana*	5	I	I	C
2000	Melastomataceae	*Miconia calophylla*	8	E	A	I
2000	Meliaceae	*Ruagea glabra*	8	I	I	I

2000	Moraceae	*Naucleopsis francisci*	9	L	C	C
2000	Myrtaceae	*Myrcia sp1*	8	L	C	I
2000	Phyllanthaceae	*Hieronyma fendleri*	9	I	I	C
2000	Podocarpaceae	*Podocarpus oleifolius*	9	L	C	C
2000	Primulaceae	*Myrsine coriacea*	8	I	I	I
2000	Rubiaceae	*Elaeagia mollis*	8	I	I	I
2000	Sapindaceae	*Matayba inelegans*	10	L	C	I
2000	Urticaceae	*Cecropia angustifolia*	10	E	A	I
3000	Aquifoliaceae	*Ilex laurina*	8	L	C	C
3000	Araliaceae	*Oreopanax sessiliflorus*	8	I	I	I
3000	Asteraceae	*Critoniopsis pycnantha*	8	E	A	I
3000	Chloranthaceae	*Hedyosmum purpurascens*	8	E	A	U
3000	Clethraceae	*Clethra revoluta*	8	I	I	C
3000	Clusiaceae	*Clusia elliptica*	8	L	C	I
3000	Cunoniaceae	*Weinmannia elliptica*	8	L	C	C
3000	Lauraceae	*Aiouea dubia*	8	L	C	C
3000	Lauraceae	*Ocotea rotundata*	8	L	C	C
3000	Melastomataceae	*Miconia cf jahnii*	8	E	A	I
3000	Siparunaceae	*Siparuna muricata*	8	E	A	U
3000	Theaceae	*Gordonia fruticosa*	9	L	C	C

Appendix A3: Phylogenetic tree of the study species.

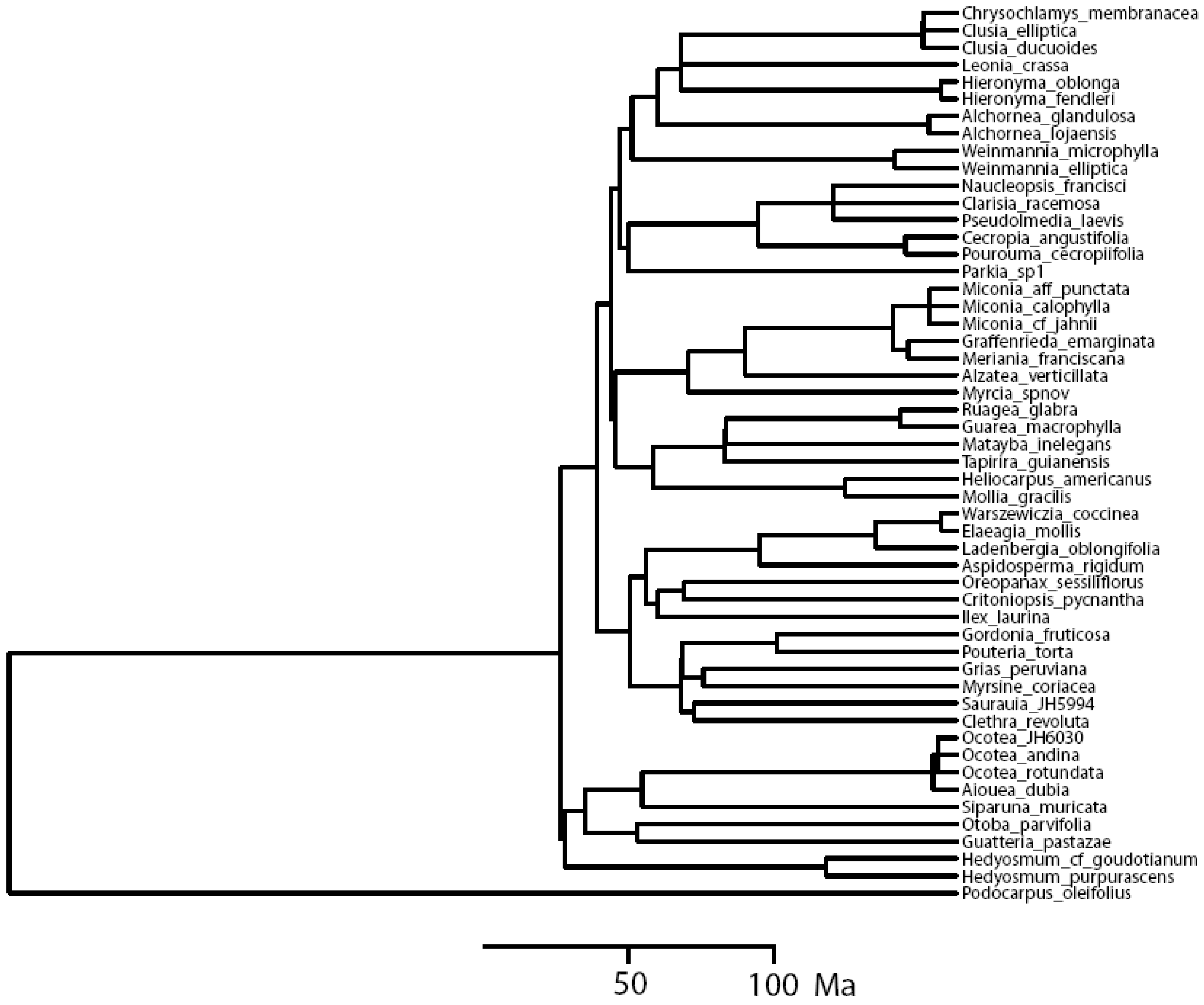

Appendix A4: Model results. Parameter estimates, estimate errors, highest density intervals (HDI), Rhat, Bulk effective sample size (ESS) and Tail effective sample size are given for all estimated parameters of twelve phylogenetic mixed models describing the effect of site on leaf traits. Parameters describing standard deviations of random intercept and the residual standard deviation are labelled sd_. The site coefficients for Ca and Bo describe the mean differences in leaf traits at the sites Cajanuma (3000 m) and Bombuscaro (1000 m) compared to the site San Francisco (2000 m). Site coefficients credibly different from zero are bold.

	**Estimate**	**Est.Error**	**HDI_low**	**HDI_high**	**Rhat**	**Bulk_ESS**	**Tail_ESS**
**Al**							
Intercept	-3.361	1.217	-5.824	-1.027	1	6316	6446
site_Bo	0.588	0.369	-0.162	1.308	1	7101	6778
**site_Ca**	**1.093**	**0.421**	**0.265**	**1.93**	1	7345	6932
sd_plot	0.263	0.142	0.028	0.535	1.003	3863	4550
sd_species	0.519	0.186	0.132	0.89	1.001	1680	1672
sd_phylo	1.935	0.318	1.343	2.579	1.001	3462	5187
sd_residual	0.748	0.029	0.693	0.806	1.001	15451	7112
**Leaf area**							
Intercept	3.284	1.221	0.885	5.683	1.001	4336	5010
**site_Bo**	**0.756**	**0.229**	**0.281**	**1.182**	1.001	5454	5323
**site_Ca**	**-1.295**	**0.28**	**-1.832**	**-0.717**	1.001	5952	5994
sd_plot	0.055	0.047	0	0.141	1.001	4054	5331
sd_species	0.285	0.206	0	0.674	1.01	634	1047
sd_phylo	1.965	0.336	1.289	2.641	1.004	1291	1338
sd_residual	0.321	0.013	0.297	0.346	1.001	15237	6532
**Ca**							
Intercept	1.233	0.411	0.404	2.089	1	9083	5397
**site_Bo**	**0.827**	**0.24**	**0.357**	**1.317**	1	7160	6655
site_Ca	0.442	0.259	-0.061	0.968	1	8081	6660
sd_plot	0.202	0.091	0.074	0.38	1	5972	7154
sd_species	0.405	0.095	0.219	0.582	1.003	1554	4139
sd_phylo	0.536	0.235	0.012	0.915	1.004	1110	1970
sd_residual	0.343	0.013	0.318	0.368	1	12669	7380
**DMC**							
Intercept	5.86	0.22	5.431	6.302	1	6349	6321
site_Bo	-0.056	0.061	-0.17	0.07	1.001	7484	6830
site_Ca	0.105	0.069	-0.03	0.242	1	7993	7119
sd_plot	0.033	0.022	0	0.073	1.001	3734	5059
sd_species	0.089	0.044	0	0.164	1.005	907	1524
sd_phylo	0.335	0.075	0.188	0.483	1.001	1437	1566

	Estimate	Est.Error	HDI_low	HDI_high	Rhat	Bulk_ESS	Tail_ESS
sd_residual	0.145	0.005	0.135	0.156	1	14641	6673
**K**							
Intercept	2.135	0.449	1.225	3.008	1.001	4434	5764
site_Bo	-0.183	0.259	-0.705	0.313	1	5197	5119
site_Ca	-0.233	0.268	-0.746	0.323	1.001	4260	4971
sd_plot	0.25	0.115	0.086	0.485	1	3859	5752
sd_species	0.168	0.099	0	0.342	1.002	783	1328
sd_phylo	0.634	0.151	0.333	0.934	1.001	1258	1314
sd_residual	0.431	0.016	0.4	0.464	1	12321	7101
**Mg**							
Intercept	0.918	0.436	0.054	1.797	1.001	8244	6991
site_Bo	0.051	0.173	-0.281	0.39	1	7830	5570
site_Ca	-0.076	0.193	-0.443	0.299	1	6985	5534
sd_plot	0.149	0.074	0.057	0.283	1	5257	5752
sd_species	0.221	0.085	0.065	0.411	1.004	1137	1754
sd_phylo	0.637	0.159	0.308	0.949	1.004	1382	1446
sd_residual	0.251	0.009	0.232	0.269	1	12985	7202
**N**							
Intercept	2.721	0.287	2.137	3.285	1.001	5117	5561
**site_Bo**	**0.22**	**0.083**	**0.059**	**0.388**	1	6105	5953
site_Ca	-0.155	0.097	-0.351	0.03	1	5575	6668
sd_plot	0.055	0.027	0.015	0.107	1	4315	5687
sd_species	0.134	0.051	0.033	0.237	1.002	1103	1606
sd_phylo	0.432	0.098	0.245	0.625	1.001	1429	1658
sd_residual	0.117	0.004	0.109	0.125	1	14807	7890
**N:P**							
Intercept	2.764	0.137	2.485	3.035	1.001	6376	5534
site_Bo	0.161	0.126	-0.075	0.413	1	5477	5479
site_Ca	0.035	0.131	-0.217	0.292	1.001	5589	5429
sd_plot	0.129	0.056	0.051	0.234	1	4759	5483
sd_species	0.145	0.03	0.085	0.204	1.003	1221	1725
sd_phylo	0.134	0.082	0	0.281	1.005	660	1641
sd_residual	0.132	0.005	0.122	0.142	1.001	15539	7456
**P**							
Intercept	-0.029	0.365	-0.781	0.666	1.001	4470	5159
site_Bo	0.028	0.162	-0.293	0.338	1	4530	5582
site_Ca	-0.196	0.168	-0.507	0.142	1	4880	5380
sd_plot	0.17	0.076	0.072	0.309	1.001	4902	5541
sd_species	0.064	0.048	0	0.154	1.007	609	1169
sd_phylo	0.561	0.075	0.421	0.716	1.003	1774	2040
sd_residual	0.172	0.006	0.16	0.185	1.001	13140	7713
**SLA**							
Intercept	4.269	0.217	3.827	4.709	1.001	7564	6097
**site_Bo**	**0.449**	**0.142**	**0.174**	**0.702**	1.001	5301	5632
**site_Ca**	**-0.294**	**0.152**	**-0.588**	**-0.011**	1	5446	5791

	Estimate	Est.Error	HDI_low	HDI_high	Rhat	Bulk_ESS	Tail_ESS
sd_plot	0.121	0.062	0.043	0.224	1.001	4838	4968
sd_species	0.207	0.044	0.122	0.294	1.002	1805	3891
sd_phylo	0.277	0.108	0.033	0.467	1.003	1364	1630
sd_residual	0.164	0.006	0.153	0.176	1	12268	7177
**Leaf thickness**							
Intercept	-1.166	0.191	-1.526	-0.757	1.001	10439	7284
**site_Bo**	**-0.363**	**0.11**	**-0.582**	**-0.146**	1	7611	6948
site_Ca	0.242	0.126	-0.008	0.486	1	7257	6588
sd_plot	0.08	0.036	0.031	0.151	1	5248	7166
sd_species	0.238	0.04	0.164	0.317	1.001	2262	4158
sd_phylo	0.246	0.107	0.004	0.415	1.002	1224	1804
sd_residual	0.12	0.005	0.111	0.129	1.001	12461	5910
**Leaf toughness**							
Intercept	0.171	0.477	-0.782	1.135	1.001	4955	5287
**site_Bo**	**-0.316**	**0.137**	**-0.578**	**-0.049**	1.001	4134	6052
site_Ca	0.227	0.153	-0.081	0.525	1.001	7206	7166
sd_plot	0.078	0.037	0.024	0.15	1.001	3918	5509
sd_species	0.224	0.101	0.015	0.413	1.004	731	1108
sd_phylo	0.716	0.193	0.342	1.101	1.003	892	1066
sd_residual	0.133	0.005	0.123	0.142	1	11840	6974

Appendix A5: Intraspecific trait variation at the three study sites, tree species are ordered by their mean values and genus names of species with high or low values are given.

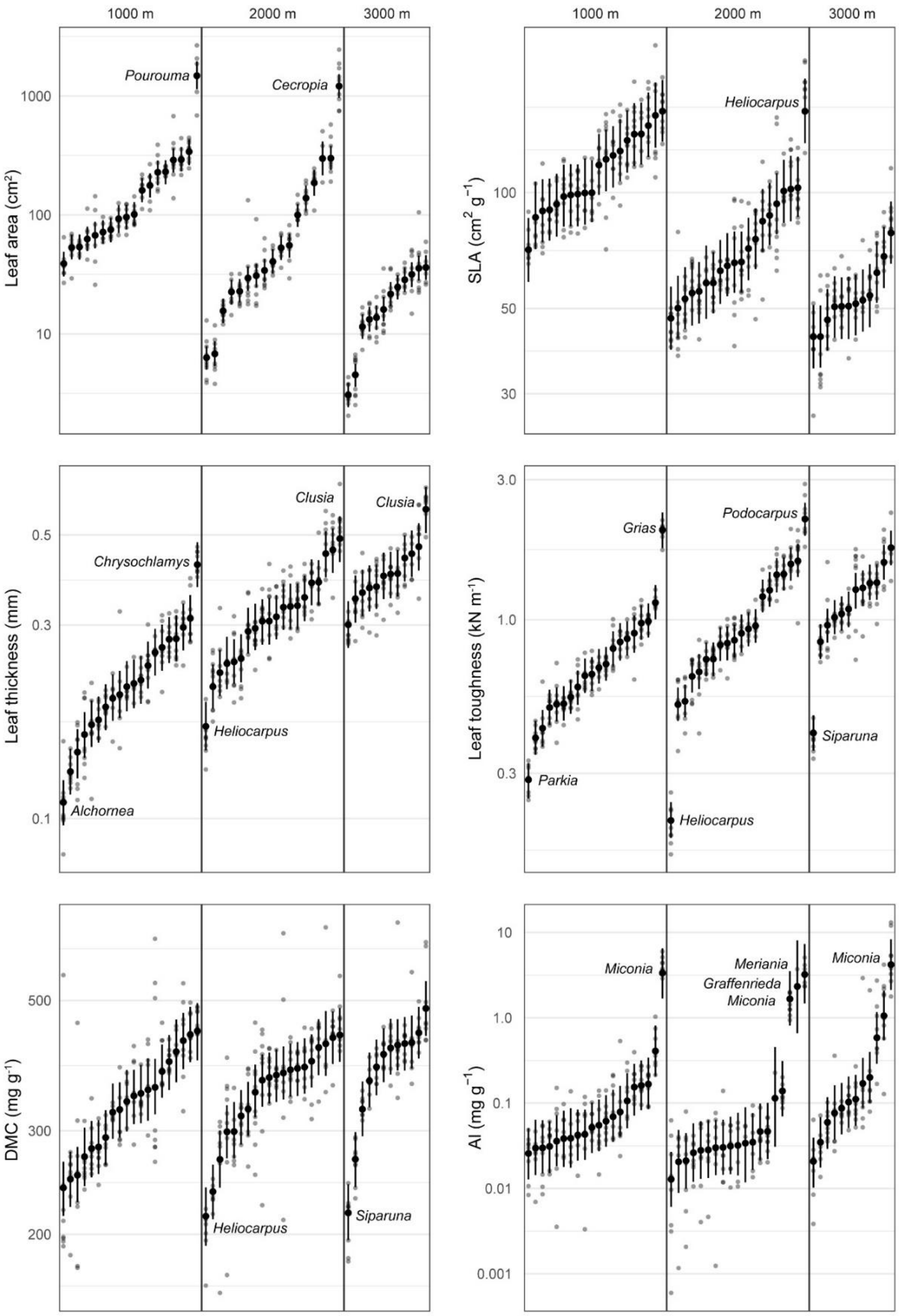

Appendix A5: continued

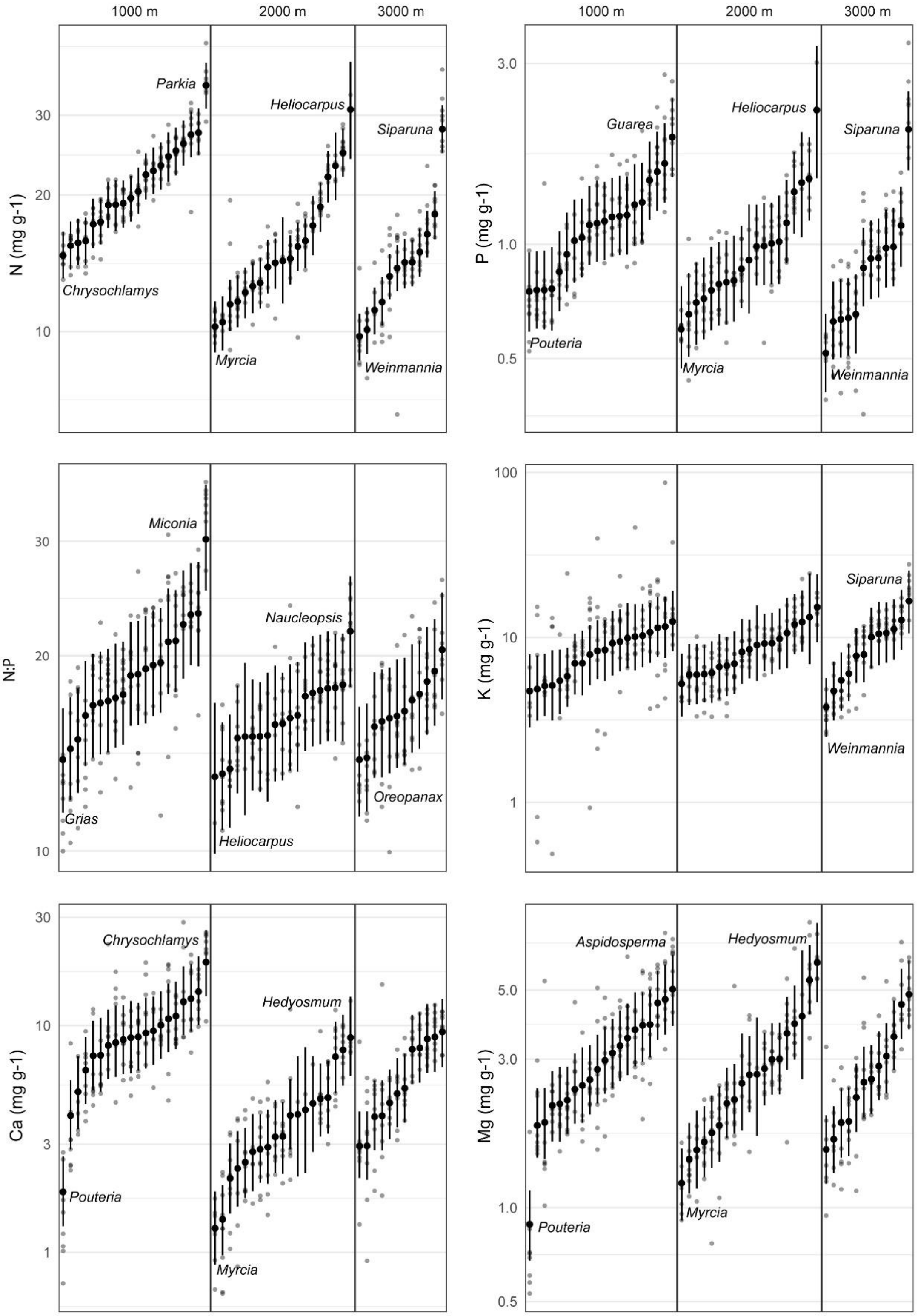

Appendix A6: Results of functional diversity partitioning into between- and within-species effects (Figure 4).

	1000m		2000m		3000m	
**Leaf area**	**funct. diversity**	**%**	**funct. diversity**	**%**	**funct. diversity**	**%**
Between species	0.0029	72.28	0.0115	85.25	0.0253	81.01
Within species	0.0011	27.72	0.0020	14.75	0.0059	18.99
Total	0.0040		0.0135		0.0312	
**SLA**						
Between species	0.0322	60.27	0.0131	63.40	0.0113	55.91
Within species	0.0212	39.73	0.0075	36.60	0.0089	44.09
Total	0.0534		0.0206		0.0201	
**Leaf thickness**						
Between species	0.0292	85.43	0.0205	76.27	0.0203	59.45
Within species	0.0050	14.57	0.0064	23.73	0.0138	40.55
Total	0.0341		0.0269		0.0341	
**Leaf toughness**						
Between species	0.0123	90.01	0.0230	90.26	0.0123	61.76
Within species	0.0014	9.99	0.0025	9.74	0.0076	38.24
Total	0.0136		0.0254		0.0200	
**DMC**						
Between species	0.0158	39.32	0.0088	39.39	0.0393	61.57
Within species	0.0244	60.68	0.0136	60.61	0.0246	38.43
Total	0.0401		0.0224		0.0639	
**Foliar N**						
Between species	0.0253	86.46	0.0212	90.39	0.0128	78.76
Within species	0.0040	13.54	0.0023	9.61	0.0035	21.24
Total	0.0293		0.0235		0.0163	
**Foliar P**						
Between species	0.0205	65.40	0.0063	77.52	0.0082	75.86
Within species	0.0108	34.60	0.0018	22.48	0.0026	24.14
Total	0.0314		0.0081		0.0109	
**Foliar N:P**						
Between species	0.0155	56.78	0.0168	61.68	0.0208	49.85
Within species	0.0118	43.22	0.0104	38.32	0.0210	50.15
Total	0.0274		0.0273		0.0418	
**Foliar Al**						
Between species	0.0070	92.68	0.0509	92.38	0.0097	58.79
Within species	0.0006	7.32	0.0042	7.62	0.0068	41.21
Total	0.0076		0.0551		0.0165	
**Foliar Ca**						
Between species	0.0163	56.43	0.0201	62.31	0.0283	67.64
Within species	0.0126	43.57	0.0121	37.69	0.0135	32.36
Total	0.0289		0.0322		0.0418	
**Foliar K**						
Between species	0.0017	20.20	0.0096	74.09	0.0258	85.88
Within species	0.0068	79.80	0.0034	25.91	0.0042	14.12
Total	0.0086		0.0130		0.0301	
**Foliar Mg**						
Between species	0.0322	76.19	0.0227	75.75	0.0333	75.94
Within species	0.0101	23.81	0.0072	24.25	0.0106	24.06
Total	0.0423		0.0299		0.0439	

Appendix A7: PCA results (Figure 4).

Importance of components

	PC1	PC2	PC3	PC4	PC5
**Standard deviation**	22.309	14.037	11.153	0.99531	0.9340
**Proportion of Variance**	0.4147	0.1642	0.1037	0.08255	0.0727
**Cumulative Proportion**	0.4147	0.5789	0.6826	0.76515	0.8378

Factor loadings

	PC1	PC2	PC3	PC4	PC5
**LA**	0.106	-0.089	0.273	-0.870	0.289
**SLA**	0.375	-0.243	-0.001	0.275	0.111
**thickness**	-0.295	0.414	-0.251	-0.013	-0.035
**toughness**	-0.311	0.316	0.079	0.020	-0.010
**DMC**	-0.289	-0.309	0.286	0.016	-0.193
**N**	0.395	-0.164	0.087	0.058	-0.167
**P**	0.385	0.169	0.234	-0.007	-0.293
**N:P**	-0.052	-0.581	-0.339	0.059	0.103
**Al**	-0.115	-0.202	-0.514	-0.375	-0.617
**Ca**	0.238	0.100	-0.461	-0.096	0.432
**K**	0.341	0.206	0.045	-0.090	-0.416
**Mg**	0.296	0.282	-0.346	-0.035	0.038

Appendix A8: Network characteristics as indicators of network centrality for the 12 studied leaf traits at the three study sites.

	**1000m**	**2000m**	**3000m**
**Degree**			
Leaf area	0	0	3
SLA	4	7	7
Thickness	3	6	4
Toughness	2	8	6
DMC	2	7	6
N	4	7	7
P	3	8	8
N:P	2	1	3
Al	1	0	0
Ca	1	3	2
K	1	7	9
Mg	3	8	7
**Node Strength**			
Leaf area	0	0	23
SLA	24	51	54
Thickness	16	34	26
Toughness	12	44	40
DMC	13	42	40
N	25	48	54
P	19	52	60
N:P	13	5	23
Al	7	0	0
Ca	6	17	14
K	5	48	65
Mg	18	47	47
**Betweenness**			
Leaf area	0	0	0
SLA	0.37	0	0
Thickness	0.44	0	0
Toughness	0	0.10	0.02
DMC	0.38	0	0.07
N	0.05	0	0.01
P	0.29	0.15	0.09
N:P	0.16	0	0
Al	0	0	0
Ca	0	0	0
K	0	0	0.23
Mg	0.31	0.02	0.07
